# Maintenance of physical activity gains among Latina teens: 12-month findings from the *Chicas Fuertes* randomized controlled trial

**DOI:** 10.1186/s12966-026-01933-w

**Published:** 2026-05-29

**Authors:** Esmeralda Castro, Jacob Carson, Emily Greenstadt, Brittany Olivera, Shira Dunsiger, Michael Higgins, Job Godino, Bess Marcus, Dawn Meyer, Michelle Zive, Britta Larsen

**Affiliations:** 1 Department of Family Medicine, University of California San Diego, 9500 Gilman Dr., La Jolla, CA USA; 2 Herbert Wertheim School of Public Health & Human Longevity Science, UC San Diego, CA La Jolla, USA; 3 Department of Behavioral and Social Sciences, Brown University, Providence, RI USA; 4 Exercise and Physical Activity Resource Center, UC San Diego, CA La Jolla, USA; 5 Laura Rodriguez Research Institute, Family Health Centers of San Diego, CA San Diego, USA; 6 Department of Neurosciences, School of Medicine, UC San Diego, CA La Jolla, USA

**Keywords:** Physical activity, Latina, Adolescents, mHealth, Health inequity

## Abstract

**Background:**

Only about three percent of Latina teens are sufficiently active. As Latinas have a higher prevalence of lifestyle-related chronic health conditions, such as diabetes, compared to non-Latinas, targeted interventions that increase and maintain moderate-to-vigorous-intensity physical activity (MVPA) are needed. Chicas Fuertes is a 12-month multi-channel mHealth physical activity intervention for Latina teens shown to increase MVPA over the first six months. The purpose of this study was to test the effectiveness of Chicas Fuertes for maintaining Latina teens’ initial physical activity gains over the subsequent six months.

**Methods:**

In a randomized controlled trial, Latina teens, aged 13 –18 and underactive, participated in Chicas Fuertes or served as a control. The intervention group initially received a Fitbit, a one-on-one coaching session and two check-in calls, access to a personalized website, tailored text messages, and daily Instagram content while the control group received only a Fitbit. From six to 12-months, the intervention group received a short coaching call and continued access to the tailored digital media content. Weekly minutes of MVPA were assessed at baseline, six, and 12-months with hip-worn Actigraph GT3X+ accelerometers and the 7-Day Physical Activity Recall Interview. Mixed effects regressions were conducted to examine between-group differences in MVPA at twelve months, with models of accelerometer data adjusting for device wear-time.

**Results:**

Of the 160 participants (Mage= 15.9 ± 1.6),137 completed the 12-month follow-up. More than half (69%) identified as second-generation and reported a household income of ≤$50,000 (62%). Weekly minutes of MVPA gained at six months were maintained at twelve months in Chicas Fuertes participants compared to control. Intervention participants had increased median changes in accelerometry-measures, 10.00 (IQR=13), compared to decreases observed in the control, -5 (IQR=21). Median changes in self-reported measures 13 (IQR=32) among intervention compared to 5 (IQR=26) among control.

**Conclusions:**

Chicas Fuertes was able to support maintenance of objective and self-reported MVPA gains from six months to twelve months with tapered support. Digital technologies that Latina teens commonly use can be leveraged to increase dissemination and implementation of physical activity interventions at a relatively low cost, ultimately enhancing health equity among Latina teens.

**Trial registration:**

ClinicalTrials.gov NCT04190225. Registered on November 20, 2019.

## Background

Engaging in physical activity (PA) can provide a host of benefits for individual health, from improved cardiorespiratory fitness [1] to reduced anxiety and depressive symptoms [2, 3]. Substantial research among adolescents also supports PA engagement for enhanced academic performance [4], self-esteem [3], and sleep [5]. However, most adolescents are insufficiently active, with adolescent girls far less likely to engage in moderate-to-vigorous physical activity (MVPA) [6] and also experience greater declines in MVPA per year [7] than adolescent boys.

Among adolescents, racial and ethnic minority girls report the lowest levels of regular PA, with Hispanic/Latina girls reporting the lowest [8]. While some previous research has focused on interventions to promote PA among adolescent girls [9], few have targeted Latina teens [10]. With cardiovascular disease and cancer as the leading causes of death among Latina women [11], regular PA can help reduce morbidity and mortality in this population. Early prevention efforts to promote PA in Latina teens are especially critical as engagement in PA during adolescence predicts PA during adulthood [12]. Research has shown that Latina teens have a strong preference for interventions that are specific to them [13], highlighting the need to develop PA interventions tailored for this population.

Currently, most interventions designed to improve MVPA among adolescents have focused on initiating PA and short-term outcomes. Few intervention studies among adolescent girls have evaluated longer-term outcomes or maintenance of PA, which is essential for promoting life-long health and preventing chronic disease [14, 15]. To date, no study has evaluated PA maintenance specifically among Latina teens. Identifying interventions that promote PA among Latina teens, are easy to deliver, and can sustain initial increases in PA engagement are needed to address disparities in life course diseases [12].

To address gaps in the literature and growing disparities, Chicas Fuertes [16] was a fully powered randomized trial of a digital multimedia MVPA intervention for Latina teens. Short-term results showed that intervention participants significantly increased objectively- and subjectively measured MVPA over the six-month active intervention period and did so significantly more than control participants [17]. Over the next six months, participants continued to receive tapered intervention doses. The purpose of the current study was to evaluate the effectiveness of the intervention on maintaining the MVPA increases with tapered support among Latina teens. It was hypothesized that participants in Chicas Fuertes would maintain the objectively and subjectively measured MVPA gains from the initial six months of the study and would engage in more time in MVPA during the final six months than the control group.

## Methods

### Study population and recruitment

Chicas Fuertes was designed for 13–18-year-old teens who identified as Latina, were underactive (i.e., reporting < 150 min of weekly MVPA), could read, write, and speak English, and resided in San Diego County. Inclusion criteria also required access to a mobile phone capable of receiving text messages and regular Internet access (i.e., ≥ 2 times/week). Exclusion criteria included preexisting health conditions that precluded engaging in unsupervised exercise, such as a serious heart condition, chest pain during exercise, and uncontrolled asthma. Recruitment occurred from March 2020 through June 2023, primarily through social media advertisements and local school presentations. Facebook and Instagram advertisements accounted for approximately 65% of total study contacts with almost 30% coming from individuals that had attended a school presentation. Remaining contacts were generated by study promotion from community partners, research repositories like clinicaltrials.gov, and word of mouth. Further details on recruitment, randomization, blinding, and baseline and 6-month activities have been described elsewhere [13, 16, 18].

### Intervention design

Chicas Fuertes is a theory-based, individually tailored, 12-month multi-technology intervention designed to increase MVPA in Latina teens. Delivered in English, the intervention was built through iterative co-design and beta testing with the target population [13]. After enrolling, participants provided baseline measures, including online questionnaires and PA measures. The study utilized a 1:1 randomization into the intervention and control groups using REDCap clinical trials software, stratifying participants by baseline level of motivational readiness to engage in PA. Participants with friends or family members in the study were yoked (25%) to the same condition to minimize potential cross-contamination. The study was approved by the Institutional Review Board at the University of California San Diego and all participants provided informed consent (if they were 18) or assent and parental consent. The trial registration, protocol, and data analysis plan are available online (clinical trial number: NCT04190225).

### Protocols

#### Intervention group - tailored mhealth physical activity intervention

Chicas Fuertes is guided by the Social Cognitive Theory which posits that behavior is influenced by individual experiences, the actions of others, and the external environment [19]. Chicas Fuertes was based on a previous culturally tailored intervention for Latina women [20] and then modified based on qualitative work among Latina adolescents [13]. Detailed descriptions of the intervention components are reported elsewhere for baseline through the first six months [16]. The summary below focuses on the final six months. 

##### Health coaching session

Within a four-week window before to after their six-month enrollment date, participants had a single, 20–30 min, 1:1 coaching session over Zoom with the interventionist from their baseline visit that emphasized reviewing lessons, assessing progress, and revising goals as needed. The intention of the session was to reinforce behavior change concepts and support participants with the necessary tools (e.g., goal setting and self-monitoring) to continue setting personalized PA goals independently. The interventionist was a non-Hispanic White woman who had an MPH degree and was a certified health coach.

##### Text messages 

Participants received tailored text messages approximately four times each week throughout the entire study period. Text messages were individually tailored based on real-time Fitbit data to update participants on their weekly goals and suggest behavior change techniques for those not on track to meet goals. Friends and family names, and favorite places to be active, were incorporated in text messages for additional personalized motivation. Messages also reminded participants to complete intervention-related tasks, such as planning their weekly activity or completing questionnaires. 

##### Study website

Participants had access to the study website throughout the entire study period, which contained a tool to schedule weekly activity and an opportunity to review their previous recorded Fitbit activity. The website provided resources for overcoming common PA barriers, links to free exercise videos, a comprehensive list of free and low-cost places to be active within the county, an interactive message board, and a leaderboard displaying the three most active participants that week. Every other month, participants completed online questionnaires on activity perceptions and behavior. These responses were used to generate an individually tailored report on their progress and strategies to try to increase their activity. To enhance adherence to website engagement, participants received compensation for completing questionnaires, and received points for using website features, which could be redeemed for prizes (e.g., water bottle, Fitbit bands). 

##### Social media

A private Instagram account was updated with daily posts integrating behavior change techniques and constructs of the Social Cognitive Theory [19]. Interactive Instagram stories allowed participants to take mini quizzes or vote in PA-related polls for additional engagement, as well as served as reminders for wearing and syncing their Fitbit. Participants were invited to follow the account but was not required. For those who chose not to follow on Instagram, social media posts could be seen on the study website.

#### Control group - fitbit only

Participants in the control group were instructed to wear, sync, and charge their Fitbit Inspire HR consistently over the 12 months. Participants received text message reminders to sync and charge their Fitbit if the device had not been synced for five days. They were able to view their Fitbit data on the watch and the app, similar to the intervention group. No additional instructions or support were provided, aside from addressing any Fitbit malfunctions.

### Measures

#### ActiGraph accelerometer

Hip-worn ActiGraph GT3X+ accelerometers (ActiGraph, LLC; Pensacola, FL) were used to objectively assess participant MVPA at baseline, six, and 12-months. Accelerometers have been validated for measuring activity intensity among adolescents [21, 22]. Participants were mailed the accelerometer with instructions to wear it on their left hip for at least 12 h a day, over seven days. The accelerometer was returned with a prepaid envelope. Valid wear time was defined as ≥ 600 min per day for five days or ≥ 3000 min across four days and was identified with the Choi algorithm [23]. Cut-points were derived from Treuth et al. [24], as these were developed and validated in adolescent girls specifically. 

#### 7-Day PAR interview

The 7-Day Physical Activity Recall (PAR) Interview is a semi-structured interview that assesses the frequency, duration, and intensity of MVPA [25]. It has been validated among adolescent girls and Latinos and shown strong validity, consistency, and sensitivity to changes over time [26, 27]. After accelerometer wear, participants met with a research staff member who had been certified in conducting the 7-Day PAR. This staff member did not participate in the health coaching sessions. The baseline visit was conducted at a local park to demonstrate moderate intensity activity through a walk, while follow up visits occurred online via Zoom video. Participants were asked to break down their PA by day, time, intensity, and purpose. To assist with recall, the interviewer provided examples of MVPA activities to help participants understand what qualified as moderate intensity.

#### Psychological constructs

The online REDCap questionnaire administered before baseline included additional measures for psychological constructs that have been validated among youth. The 5-item Self-Efficacy for Physical Activity scale assessed participants’ confidence in becoming physically active [28]. The 13-item Social Support for Exercise scale assessed participants’ social support for exercise over the last three months [29]. The 18-item Physical Activity Enjoyment Scale measured personal satisfaction with PA [30]. The Neighborhood Environment Walkability Scale for Youth [31] and the 9-item Physical Activity Neighborhood Environment Scale assessed participants’ neighborhood environment [32]. Finally, participants completed the 10-item Center for Epidemiological Studies Depression Scale to evaluate depressive symptoms over the past week [33].

### Statistical analyses

Although presented elsewhere, a summary of baseline socio-demographics and PA behavior were summarized in the aggregate sample and between-groups using means and standard deviations (for continuous variables) and frequencies (percent) for categorical variables. All analyses were run in R version 4.4.1 with significance level set at 0.05 a priori. As min/week of MVPA was skewed, a series of quantile regression models with bootstrapped standard errors (10,000 bootstrapped samples) were used to examine between-group differences in self-reported and device-measured min/week of MVPA at twelve months adjusting for previous time periods (baseline and six months). Models included the main effects of randomization (intervention vs. control), time, group*time, as well as covariates including an indicator of whether participants were yoked together at randomization, and in the case of accelerometer-measured outcomes, device wear-time.

The effects of randomization at six months have been presented [17]. Thus, time effects were specified (with contrasts) to capture between-group differences from six to 12 months, adjusting for initial uptake in activity baseline to six months. Models were run separately for self-reported MVPA (via 7-day PAR) and device-collected min/week MVPA (via accelerometer). Although analysis was run on the intent-to-treat sample, device-measured models were restricted to those with valid wear-time (88% of the sample). As the trial was determined to be low risk, participants were asked to self-report adverse events, and no stopping guidelines were used. No adverse events were reported.

## Results

### Study retention

**Fig. 1:**
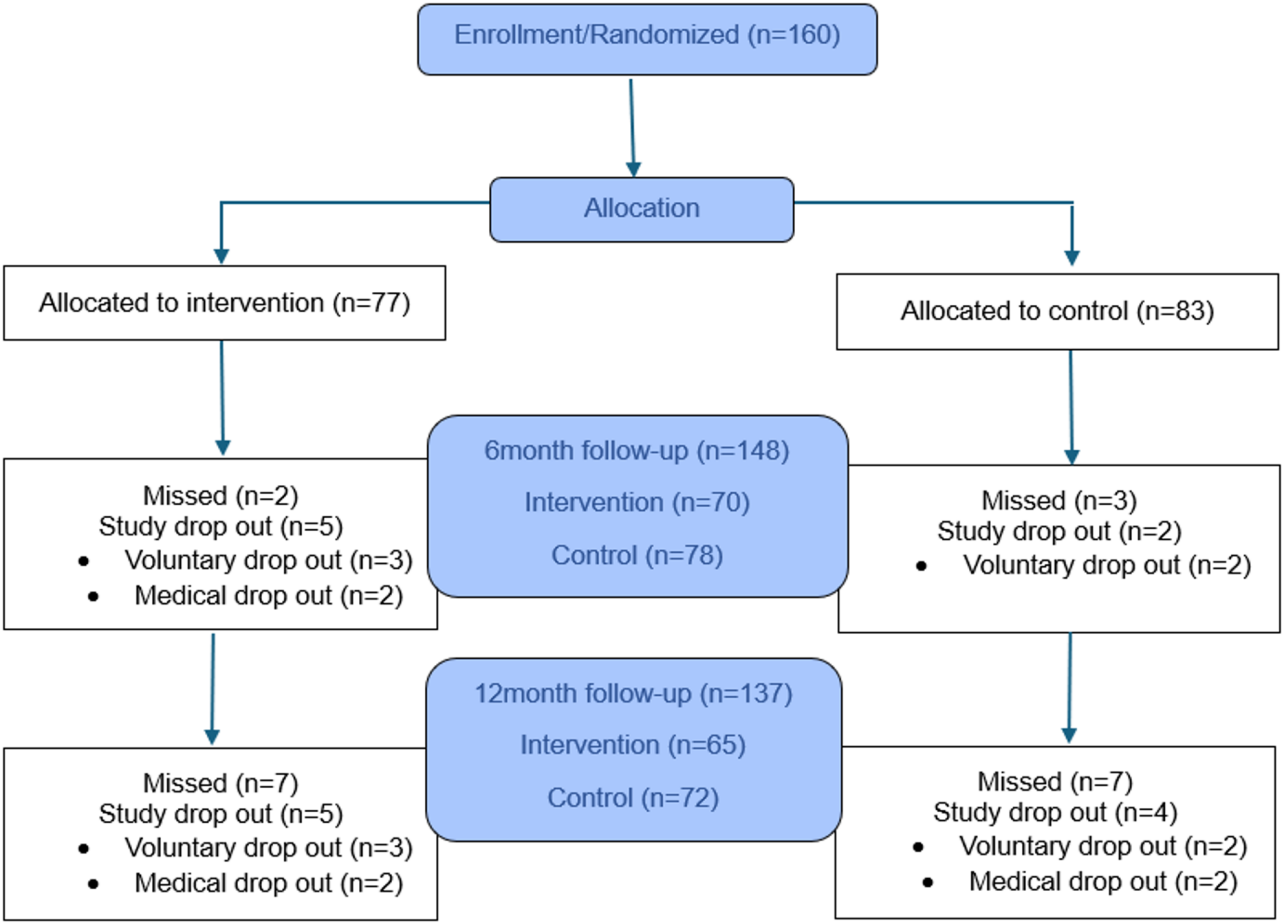
CONSORT diagram for study period. Note: Total participant numbers at 6- and 12-months are not mutually exclusive as participants may have missed the 6-month follow-up but completed the 12-month follow-up. Follow-ups missed were due to non-responders. Drop out numbers at 12 months include those reported at six months

In total, 160 Latina teens were enrolled in the study, with 77 participants being randomized to participate in Chicas Fuertes. About 93% of participants returned for the 6-month follow up and 86% returned for the final, 12-month follow up. A CONSORT diagram illustrating participants through the study period is provided in Fig. 1.

### Study sample

The aggregate sample consisted of 160 participants with an average age of 15.9 years ± 1.6, of whom 137 (86%) completed the 12-month follow-up. Among the randomized sample, nearly half (49%) self-identified as White, 69% second-generation, and 62% reported a household income of $50,000 or below. There were no between-group differences at baseline. A complete description of the study sample is presented in Table 1.

**Table 1:** Baseline Characteristics of the Study Sample by group (N=160)

	Intervention (n=77)	Control (n=83)
Participant Characteristics		
Age (years); M (SD)	15.80 (1.71)	15.90 (1.50)
BMI; M (SD)	26.16 (5.49)	25.24 (6.57)
Race; n (%)		
White	35 (45.5%)	33 (39.8%)
Black	1 (1.3%)	0 (0.0%)
Asian	1 (1.3%)	1 (1.2%)
American Indian/Alaska Native	1 (1.3%)	2 (2.4%)
Pacific Islander/Hawaiian	0 (0.0%)	0 (0.0%)
Multiracial	8 (10.4%)	4 (4.8%)
Other	31 (40.3%)	43 (51.8%)
Primary Language; n (%)		
Spanish>English	4 (5.2%)	6 (7.2%)
Spanish and English Equally	21 (27.3%)	31 (37.3%)
English>Spanish	47 (61.0%)	37 (44.6%)
Only English	5 (6.5%)	9 (10.8%)
Generational Status; n (%)		
First Generation	6 (7.8%)	7 (8.4%)
Second Generation	53 (68.8%)	57 (68.7%)
Third Generation	18 (23.4%)	19 (22.9%)
Parent Characteristics		
Education; n (%)		
<High School	26 (33.8%)	25 (30.1%)
High School/GED	16 (20.8%)	19 (22.9%)
Some College/Associate Degree	14 (18.2%)	18 (21.7%)
College Graduate	13 (16.9%)	10 (12.0%)
Master’s Degree	8 (10.4%)	8 (9.6%)
Doctoral Degree	0 (0.0%)	3 (3.6%)
Income; n (%)		
<$11,999	12 (15.6%)	22 (26.5%)
$12,000-$24,999	12 (15.6%)	11 (14.3%)
$25,000-$49,999	21 (27.3%)	21 (27.3%)
$50,000-$99,999	21 (27.3%)	18 (21.6%)
$100,000+	11 (14.3%)	11 (14.3%)
Number of Children in Home; M (SD)	2.01 (1.18)	1.93 (1.20)
Psychosocial Constructs		
Neighborhood Environment (PACES)^1^		
Land-Use Diversity; M (SD)	2.47 (0.62)	2.37 (0.81)
Recreation; M (SD)	2.27 (0.68)	2.21 (0.76)
Residential Density; n (%)	112 (39.1)	109 (40.3)
Land-Use Access; M (SD)	2.94 (0.50)	3.01 (0.50)
Street Connectivity; M (SD)	2.83 (0.59)	2.84 (0.57)
Walk/Cycle Facilities; M (SD)	3.07 (0.59)	3.05 (0.66)
Neighborhood Aesthetics; M (SD)	2.74 (0.66)	2.89 (0.73)
Safety From Traffic; M (SD)	2.78 (0.45)	2.79 (0.45)
Safety From Crime; M (SD)	2.26 (0.78)	2.19 (0.75)
Neighborhood Environment (PANES)^2^; M (SD)	4.96 (1.16)	4.59 (1.38)
PA Enjoyment; M (SD)	4.67 (0.92)	5.03 (0.93)
Depressive symptoms; M (SD)	10.62 (3.87)	11.14 (4.70)
Exercise Self-Efficacy; M (SD)	1.98 (0.54)	2.03 (0.46)
Friend Social Support for Exercise; M (SD)	20.5 (13.4)	24.2 (16.7)
Maternal Support for Exercise	2.46 (0.75)	2.55 (0.64)
Stage of Change; n (%)		
Pre-contemplation	3 (3.9%)	1 (1.2%)
Contemplation	10 (13.0%)	12 (14.5%)
Preparation	64 (83.1%)	70 (84.3%)
Physical Activity		
MVPA (min/wk); Median (IQR)		
7-Day PAR^3^	119 (122.5)	120 (186.25)
ActiGraph GT3X+^3^	0 (26)	0 (24)
Total Steps^4^; Median (IQR)	5,196 (3,116)	5,175 (3,191)

^1^PACES: Physical Activity Enjoyment Scale. ^2^PANES: Physical Activity Neighborhood Environment Scale. ^3^Variable had a skewed distribution. ^4^Total steps derived from ActiGraphs. There were no significant between-group differences among baseline characteristics (all *p*-values <.05) based on t-tests for continuous variables, chi-squared tests for categorical variables, and Mood’s median test for medians

### Effects – Six to 12 months

**Fig. 2:**
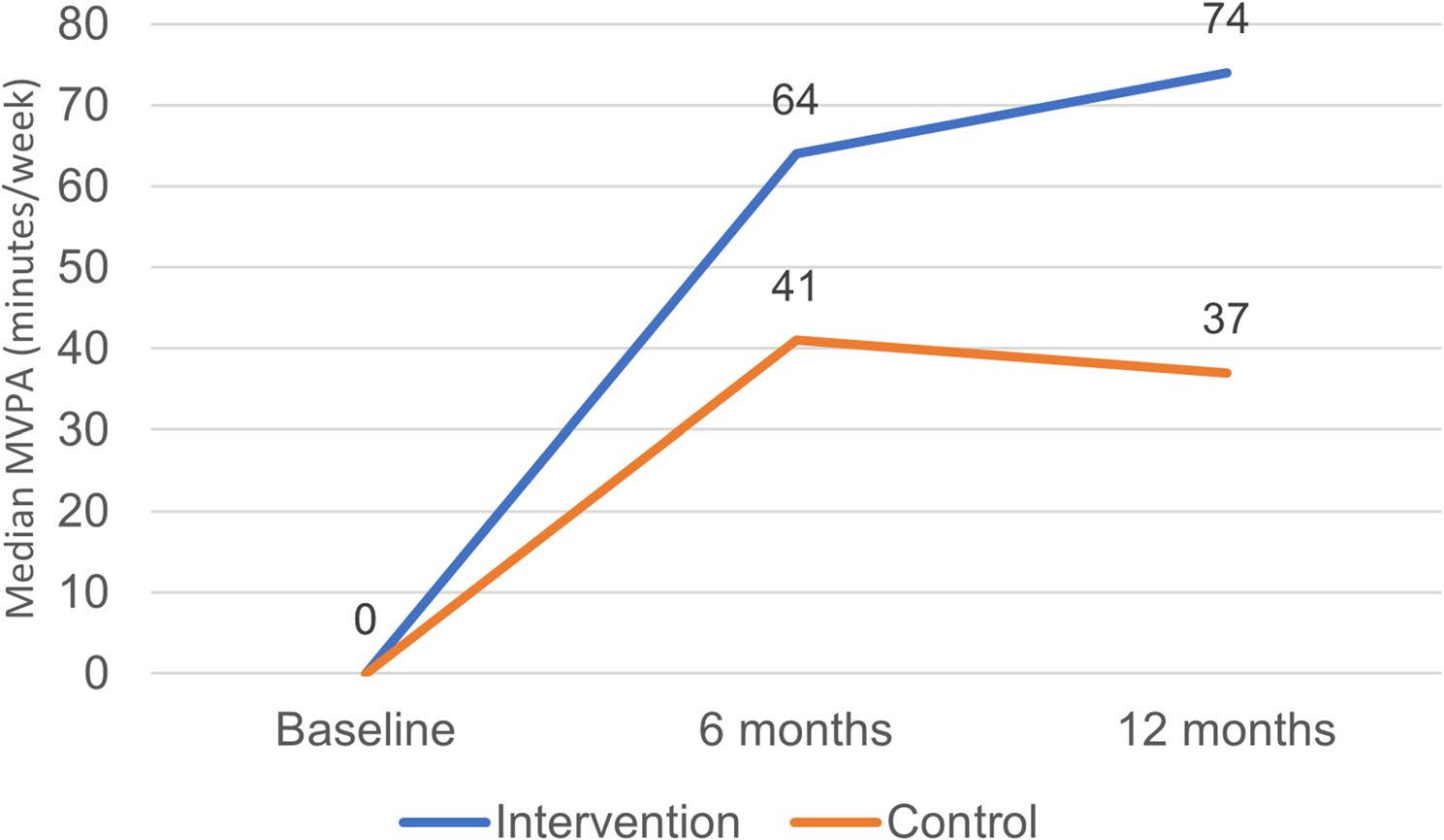
Between-group changes over time in MVPA (Accelerometer). Note: Baseline, 6-month, and 12-month visit sample sizes were 77, 70, and 65 for the Intervention Group and 83, 78, and 72 for the Control Group

Six month results of between-group differences in min/week of MVPA reported greater MVPA among intervention participants compared with control participants [17]. Models of baseline through 12-month data show that the gains achieved at six months (both objectively measured and self-reported) were maintained at 12 months. Specifically, for the accelerometer collected data (Fig. 2), MVPA from six to 12 months among intervention participants increased by 10 min/week (interquartile range (IQR) = 13), from 64 at six months to 74 at 12 months (IQR 87.5). Activity in the control group, conversely, decreased by 5 min/week (IQR = 21), from 42 to 37 (IQR = 79). Neither within-group change was significant, suggesting that the gains had been maintained. This corresponds to a nearly 15 min/week difference in median changes from six to 12 months between groups (*p* < .001).

**Fig. 3:**
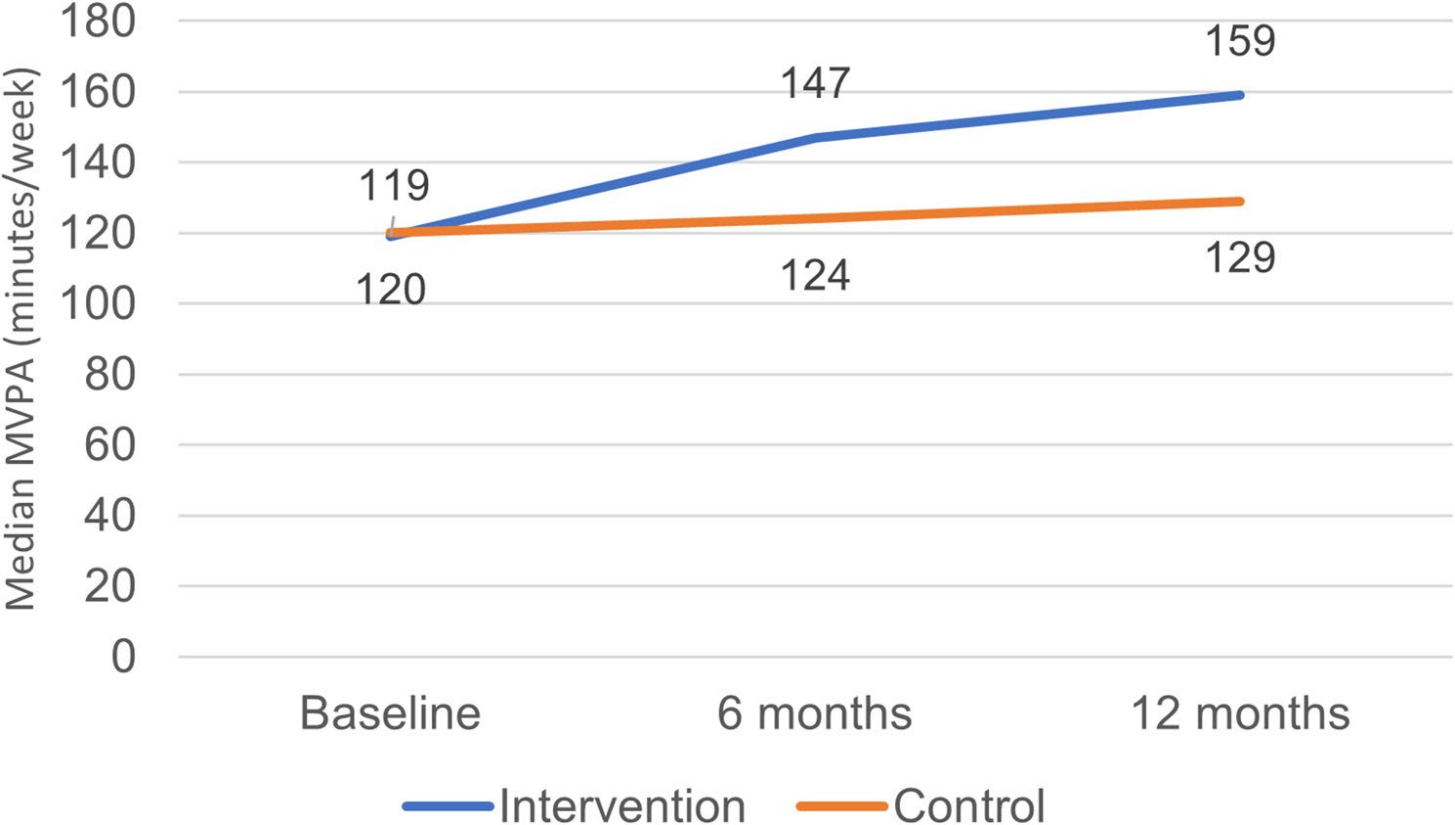
Between-group changes over time in MVPA (PAR). Note: Baseline, 6-month, and 12-month visit sample sizes were 77, 70, and 65 for the Intervention Group and 83, 78, and 72 for the Control Group

For the self-reported data (Fig. 3), MVPA from six to 12 months among the intervention participants increased by 13 min/week (IQR = 32), from 146 at six months to 159 at 12 months (IQR 122). Activity in the control group increased by 5 min/week (IQR = 26), from 124 to 129 (IQR = 148). Neither within-group change was significant The between-group difference in medians from six to 12 months was 12 min/week (*p* = .02).

## Discussion

While Hispanic/Latina teens report the lowest levels of MVPA among adolescents [8], few PA promotion interventions have targeted this group [10]. Finite trials of behavioral interventions are often short-term in research, clinical, and community settings. However, comprehension of maintained behavior change is important for life-long health and to prevent chronic health conditions. The significant findings from this study address these literature gaps, specifically for MVPA maintenance with tapered support through a digital multimedia MVPA intervention. Findings presented here are encouraging for clinicians and public health practitioners.

As hypothesized, over the final six months, Chicas Fuertes participants were able to maintain increases in MVPA made during the initial six-month intervention period, resulting in about a 15-minute difference of median changes with the control group. Findings suggest that Chicas Fuertes helped maintain increases for PA behaviors with tapered support in the final six months, as demonstrated by their accelerometer-measured increase of about ten minutes of MVPA compared to control group’s four-minute decrease. While participants did not reach the PA recommended guidelines, the increases seen in activity, specifically the 37-minute difference between groups at 12 months, suggest that Chicas Fuertes participants may have begun to form habits for PA. This is noteworthy given that participants were initially markedly inactive, with a median of zero minutes/week of objectively measured MVPA at baseline. Habit formation and skill acquisition for PA engagement is important for future behavior of PA, particularly as girls tend to participate in less PA as they get older [7]. The maintained MVPA gains after reducing the frequency of tailored feedback and one-on-one contact (the brief six-month counseling session) is also promising. As staff counseling was the most resource-demanding intervention component, positive findings seen even when this was reduced speak to the potential for scaling-up of Chicas Fuertes to larger cohorts.

The findings here are consistent with those from shorter duration interventions utilizing Fitbits in different extents (e.g., daily wear for seven days, twelve weeks) [34, 35]. The current study, the first to utilize a 12-month digital multimedia intervention to enhance Latina teens’ PA, further demonstrated the maintenance ability that interventions combined with Fitbits could provide. Current findings are also in line with prior research among first-year medical students in which Fitbits were integrated with additional intervention content (e.g., weekly reminders, group walks) and were more effective at increasing activity over 10 months than a Fitbit alone [36]. The maintained MVPA gains with tapered support among Chicas Fuertes participants could be due to the community engagement used to develop the intervention, the tailored feedback, the personalized website and social media content that could be accessed anywhere and anytime, and messaging specifically relevant to Latina teens [18]. Although previous literature has suggested longer PA trials have reduced effects due to the waning of initial interest [37], utilizing Fitbits and similar devices as a tool to reinforce theory-based approaches could support habituation.

Limited past studies have shown maintained activity over time in adolescent girls using multi-component interventions, particularly among Latinas. Maintained activity gains were seen for more than a year for non-Hispanic White and African American girls [38] and in a study of Polish adolescents [39]. However, both school-based studies were more resource intensive, relying on school staff/resources to enact changes in physical education practices, the school environment [38], and in planning out-of-school activities alongside frequent one-on-one meetings with the physical education teacher [39]. The current study provides evidence that increases in activity can also be maintained via digital channels with automated interactions and tailoring to individuals. This highlights the relative utility of comprehensive mHealth interventions in the current technological climate, allowing for rapid dissemination through multiple communication channels (i.e., websites, text messaging, social media).

### Strengths and limitations

The current study has several strengths that fill the research gap on PA maintenance among Latina teens. First, the randomized design increases the internal validity of the findings, ensuring that any confounding variables were balanced among groups. The study sample allowed for adequate power, important as previous studies have rarely considered this subpopulation for long-term PA. Relatedly, no previous PA intervention had observed Latina teens for an entire year. Another strength is the integration of both validated objective and subjective measures for MVPA. The combination of measures allows for a more comprehensive understanding of PA behaviors among participants, supporting the accuracy and validity of the findings. Intervention components, such as the one-on-one health coaching session, tailored text messages, and personalized website were specifically aimed at Latina teens, having gone through multiple iterations of co-design with the target population. This study strength addressed their specific needs and preferences, potentially increasing adherence and PA outcomes [40].

This study is not without its limitations. The findings may not be generalizable to other Latina teens as the current sample was obtained from San Diego County and consisted primarily of second-generation Latina teens of predominantly Mexican background. How the findings may have differed among first-generation Latina teens and other ethnicities, such as from the Caribbean and Central America, is unknown. Chicas Fuertes also lacked cultural tailoring of intervention components, which could have led to additional impact. The self-report 7-Day PAR and accelerometer measurements were collected at only three timepoints, at baseline, six, and 12 months. While this limits our ability to capture changes in PA trends between measurements, this sample has demonstrated agreement between accelerometers and Fitbits [41], thus future research could examine daily trajectories of activity change using data from Fitbits. This study did not assess activity post intervention and results cannot speak to sustainability of behavior changes once all active intervention components were removed. While this study focused on PA maintenance with tapered support, future studies among Latina teens should assess MVPA with post-intervention assessments, such as at 18 and 24 months, to determine long-term sustainability outcomes. Additionally, mixed methods studies would be beneficial to understand participants’ perspectives on the specific facilitators of the program for physical activity.

## Conclusion

Chicas Fuertes, a relatively low-cost, low-resource intervention tailored to Latina teens, is effective in maintaining MVPA gains over a year. As Latina teens participate in the lowest levels of PA compared to other subgroups of teens, interventions that can increase and maintain MVPA are needed. Leveraging the popularity of various digital channels among adolescents can be powerful in broadly disseminating tailored PA interventions among underactive adolescents, such as Latinas. Future studies that investigate intervention effects on MVPA behavior after program removal are needed as life-long PA habits are important for positive health outcomes.

### Data Availability

The de-identified data and statistical code supporting the conclusions of this article can be requested from the PI.

## Data Availability

The de-identified data and statistical code supporting the conclusions of this article can be requested from the PI.

## Abbreviations

PA: Physical activity
MVPA: Moderate-vigorous physical activity
PAR: Physical activity recall
IQR: Interquartile range
